# Transcriptomic Traces of Noise Exposure in Hearing Loss and Systematic Identification of Biomarker Candidates at the Molecular Scale

**DOI:** 10.3390/ijms27104182

**Published:** 2026-05-08

**Authors:** Gözde Öztan, Halim İşsever, Yahya Güldiken, Sevgi Canbaz, Fatma Oğuz, Özlem Kar Kurt, Tuğçe İşsever

**Affiliations:** 1 Department of Medical Biology, Istanbul Faculty of Medicine, Istanbul University, Topkapi, 34093 Istanbul, Turkey; oguzsf@istanbul.edu.tr; 2 Department of Public Health, Istanbul Faculty of Medicine, Istanbul University, Topkapi, 34093 Istanbul, Turkey; hissever@istanbul.edu.tr (H.İ.); sevgi.canbaz@istanbul.edu.tr (S.C.); 3 Department of Otolaryngology (Ear, Nose and Throat), Istanbul Faculty of Medicine, Istanbul University, Topkapi, 34093 Istanbul, Turkey; yahya.guldiken@istanbul.edu.tr; 4 Yedikule Chest Diseases and Thoracic Surgery Training and Research Hospital, Zeytinburnu, 34020 Istanbul, Turkey; okarkurt@gmail.com; 5 Turkish Health Institutes Presidency (TUSEB), Koşuyolu, 34718 Istanbul, Turkey; tugceissever@gmail.com

**Keywords:** noise-induced hearing loss, occupational noise exposure, peripheral blood transcriptomics, RNA sequencing, immune activation, small nucleolar RNAs, non-coding RNAs, biomarker discovery

## Abstract

Occupational noise-induced hearing loss (NIHL) is a common occupational disorder, yet non-invasive molecular indicators of chronic occupational noise exposure remain insufficiently characterized. Although the cochlear mechanisms behind NIHL have been extensively studied in experimental models, peripheral blood transcriptomic alterations in affected human populations are less well defined. In this exploratory study, we aimed to describe peripheral blood gene expression patterns associated with occupational NIHL and to generate candidate molecular signals for future validation. Peripheral blood RNA sequencing (RNA-seq) was performed in 11 male individuals with occupational bilateral sensorineural hearing loss and four noise-unexposed healthy male controls. Transcript abundance was quantified using a standardized RNA-seq workflow, and formal differential expression analysis was conducted on gene-level count data derived from Salmon quantification using DESeq2 with Benjamini–Hochberg correction. Through our analysis, we identified a limited set of differentially expressed genes, including upregulated interferon-associated transcripts, such as *RSAD2*, *IFIT1*, *IFI44L*, and *CMPK2*, host-defense-related genes, including *DEFA1*, *DEFA3*, and *DEFA4*, and immune-regulatory transcripts such as *HLA-DRB1* and *GPR15*, together with downregulated non-coding RNAs including *SNORD3A* and *SNORD3C*. These findings suggest that occupational NIHL may be accompanied by detectable peripheral blood transcriptomic alterations, predominantly involving immune- and host-defense-related pathways. Given the limited cohort size and exploratory design, these genes represent preliminary candidates for validation in larger independent cohorts.

## 1. Introduction

### 1.1. Occupational Noise Exposure and Sensorineural Hearing Loss

Occupational noise exposure remains a leading cause of acquired hearing impairment, particularly bilateral sensorineural hearing loss (SNHL), which accounts for a significant proportion of the global occupational health burden [1]. Chronic exposure to industrial noise exceeding permissible sound pressure levels leads to progressive and irreversible damage to cochlear hair cells, synaptic connections, and auditory nerve fibers. This pathology results in reduced speech discrimination, permanent threshold shifts, and increased risk of cognitive and psychological sequelae [2]. According to the World Health Organization (WHO), over 16% of disabling hearing loss worldwide in adults is attributable to occupational noise exposure [1].

Chronic exposure to sound levels exceeding 85 dB(A), a threshold defined by the National Institute for Occupational Safety and Health (NIOSH) as hazardous, leads to a range of histopathological alterations within the cochlea, including damage to outer hair cells, ribbon synapse degeneration, and neural fiber loss. Clinically, these changes manifest as elevated auditory thresholds, impaired speech discrimination in noisy environments, and, in some individuals, secondary symptoms such as tinnitus or hyperacusis [3,4]. Occupational noise-induced hearing loss not only impairs auditory sensitivity and speech comprehension but also contributes to broader psychosocial consequences such as reduced work performance, social withdrawal, and emotional distress. These outcomes, particularly when unaddressed, may indirectly impact cognitive well-being over time [3].

Furthermore, prolonged exposure to excessive noise levels not only initiates biochemical changes in the cochlea but also leads to ultrastructural damage, including cytoskeletal disorganization and synaptic degeneration within sensory hair cells. Henderson et al. demonstrated that free radical accumulation disrupts cellular architecture, damages cytoskeletal proteins, and compromises synaptic integrity, ultimately contributing to permanent sensorineural hearing loss [5]. Similarly, Fetoni et al. provided evidence that excessive mitochondrial reactive oxygen species (ROS) generation triggers apoptosis and affects mitochondrial DNA stability, identifying mitochondrial damage as a pivotal mechanism linking oxidative stress to progressive cochlear degeneration [6]. These findings converge around the notion that redox imbalance and mitochondrial dysfunction represent core pathophysiological drivers in the development of noise-induced hearing loss, reinforcing the vulnerability of cochlear structures to oxidative and metabolic disturbances under acoustic overstimulation [5,6].

Overactivation of glutamate receptors at the inner hair cell synapses has been shown to result in excessive calcium influx and excitotoxicity. This process contributes to synaptic degeneration and long-term loss of afferent auditory nerve fibers, a phenomenon referred to as synaptopathy [7]. Inflammatory cascades involving pro-inflammatory cytokines such as tumor necrosis factor-alpha (TNF-α) and interleukin-1 beta (IL-1β) have been detected in noise-exposed cochlear tissues, further exacerbating tissue damage and impairing regeneration [8]. While these mechanisms are well documented in animal studies, translational research in human populations remains limited [9].

### 1.2. Transcriptomic Approaches in Auditory Research

Recent advances in transcriptomic technologies have transformed our understanding of gene expression dynamics in auditory biology. High-throughput approaches such as RNA sequencing (RNA-seq) and microarrays have enabled the identification of differentially expressed genes, non-coding RNAs, and molecular pathways associated with hearing function and pathology. These methods have been instrumental in elucidating mechanisms underlying cochlear development, noise-induced hearing loss (NIHL), age-related hearing loss, and genetic forms of deafness [10,11].

While initial transcriptomic investigations primarily utilized cochlear tissues from animal models, peripheral blood has emerged as a promising non-invasive alternative to monitor systemic responses to environmental exposures, including noise, given its dynamic reflection of whole-body physiological states [12]. Blood-based transcriptome profiling offers the advantage of accessibility and reflects systemic inflammatory, oxidative, and immune-related changes that may mirror or precede pathological alterations in the inner ear [10,12]. This approach is particularly valuable in human studies where cochlear sampling is not feasible.

Studies have demonstrated that noise exposure alters the expression of genes involved in immune signaling, oxidative stress, synaptic function, and apoptosis, both in the cochlea and in circulating leukocytes [13,14]. Such transcriptomic signatures not only improve our mechanistic understanding of auditory damage but also provide a foundation for biomarker discovery and personalized hearing protection strategies [15]. Nevertheless, the extent to which peripheral blood transcriptomic changes persist after established occupational NIHL and remain suitable for biomarker-orientated interpretation is still not completely understood.

### 1.3. Rationale and Objectives of the Study

Despite increasing recognition of the systemic impact of chronic noise exposure, the transcriptomic alterations detectable in peripheral blood remain poorly defined. Prior studies have demonstrated that blood-based gene expression profiling can reflect systemic biological responses and may serve as a non-invasive surrogate when target tissues like the cochlea are inaccessible [12,16].

Understanding the gene expression landscape associated with occupational SNHL could allow us to identify key pathogenic mechanisms and facilitate the discovery of accessible biomarkers for early detection and monitoring. In this study, we aimed to investigate the transcriptomic profiles of individuals diagnosed with occupational noise-induced bilateral SNHL compared to healthy controls without auditory impairment.

Using Illumina RNA-seq and a robust statistical pipeline, we sought to (i) identify differentially expressed genes associated with occupational noise exposure, (ii) functionally annotate significant genes using curated biological databases, and (iii) characterize the major biological processes represented in the resulting peripheral blood transcriptomic profile. Machine-learning-based classification was incorporated as a supplementary exploratory analysis, whereas the primary analytical framework of the study was centered on transcriptomic profiling and differential expression analysis. To our knowledge, this is one of the few studies to characterize peripheral blood transcriptomic responses to chronic occupational noise exposure in humans. These findings provide preliminary insights into systemic molecular changes associated with occupational noise exposure and may inform future biomarker-orientated studies.

## 2. Results

To investigate the molecular signatures associated with occupational noise-induced SNHL, a comprehensive transcriptomic analysis was performed on peripheral blood RNA samples. Comparative analyses between SNHL patients and healthy controls included distribution assessments, unsupervised clustering, dimensionality reduction, differential gene expression testing, and functional annotation. The following subsections present the major findings of this analysis.

### 2.1. Sociodemographic and Audiometric Data in Noise-Induced Hearing Loss

The mean age of participants in the NIHL group was 48.5 ± 4.5 years, while that of the control group was 44.5 ± 4.8 years; this difference was not statistically significant (t = 1.49, *p* = 0.15). The mean duration of occupational exposure was 18.5 ± 5.7 years for the NIHL group and 16.5 ± 2.5 years for controls, with no significant difference observed (t = 0.66, *p* = 0.51). Thus, both groups were comparable in terms of age and work duration. The control group size was limited (*n* = 4), and this should be considered when interpreting between-group transcriptomic comparisons.

The 11 participants with bilateral noise-induced hearing loss worked in various sectors, including metal casting, textiles, furniture, and the automotive industry. The control group consisted of office workers who were not occupationally exposed to noise. Among the individuals with NIHL, one had a diagnosis of asthma, and the control group had no comorbidities.

Table 1 shows pure-tone hearing thresholds at multiple frequencies. Statistically significant threshold shifts were observed in the NIHL group compared to controls at key frequencies in both ears. Right- and left-ear thresholds were significantly higher at 2, 3, 4, 6, and 8 kHz (*p* < 0.05). These shifts reflect the classic “acoustic notch” pattern typically observed in NIHL, particularly at 4 and 6 kHz, where hearing loss exceeds an average of 50 dB. Although the patients were employed in different occupational settings, the audiometric phenotype converged on a broadly similar high-frequency pattern, indicating that chronic industrial noise exposure in these workers was associated with a common NIHL-like hearing profile at the group level.

**Figure 1:**
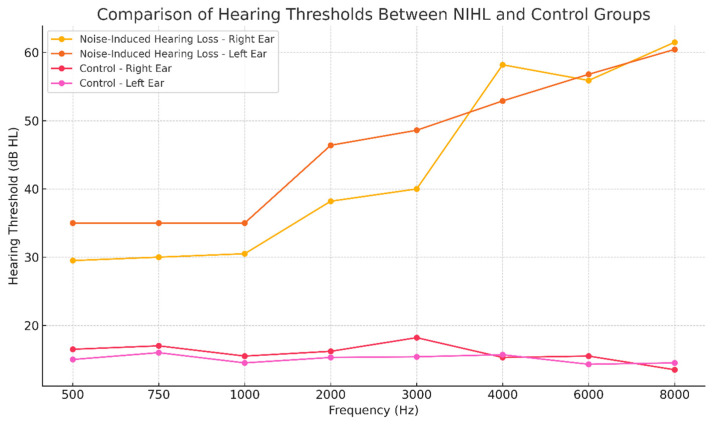
Hearing thresholds across frequencies in the noise-induced hearing loss (NIHL) and control groups. Data are presented as mean ± standard deviation (SD) for the right and left ears at each frequency (0.5–8 kHz). The NIHL group exhibited significantly elevated hearing thresholds, particularly at higher frequencies (≥2 kHz), consistent with a characteristic high-frequency hearing loss pattern. Statistical comparisons between groups were performed using the Mann–Whitney U test, and *p* < 0.05 was considered statistically significant.

Hearing thresholds were assessed across frequencies from 0.5 to 8 kHz in both NIHL and control groups. The NIHL group showed a progressive increase in threshold elevation toward the higher frequencies, with the most pronounced differences observed above 2 kHz. This distribution is compatible with the typical audiometric configuration of occupational noise-induced hearing loss and indicates greater vulnerability of the high-frequency region to chronic acoustic exposure. Although participants were exposed to different occupational noise environments, the group-level audiometric profiles converged around a broadly similar high-frequency pattern (Figure 1).

### 2.2. Sample-Level Distribution of log2(TPM + 1) Values

**Figure 2:**
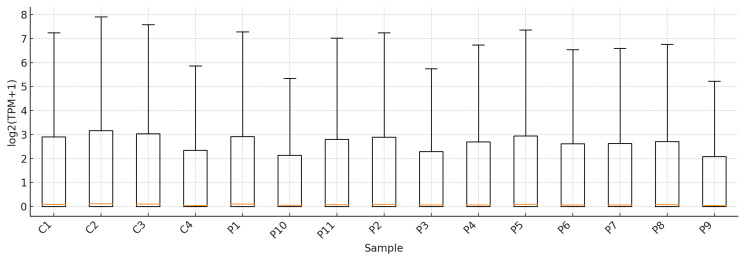
Boxplots of log2(TPM + 1) values across 15 peripheral blood RNA-seq samples. Samples are displayed individually, with controls shown first (C1–C4), followed by NIHL cases (P1–P11). In each boxplot, the red central horizontal line represents the median, the box indicates the interquartile range, and the whiskers indicate the spread of the distribution. The relatively low median values reflect the fact that a large proportion of genes had low expression values across samples after log2(TPM + 1) transformation. The figure is presented as a descriptive overview of sample-level expression distributions.

The boxplot of log2-transformed log2-transformed transcripts per million (TPM) values provides a descriptive overview of sample-level expression distributions across the 15 peripheral blood RNA-seq samples (Figure 2). The low position of the median line across samples is attributable to the large number of genes with low TPM values, even after log2(TPM + 1) transformation. The median expression levels appeared broadly comparable across samples, although variation in interquartile range and whisker length was observed among individual cases and controls. These differences should be interpreted cautiously, because TPM-based distributions may reflect normalization effects and differences in sample composition in addition to biological variability. Accordingly, the boxplot is to be used as a descriptive visualization of sample-level expression patterns and not as formal evidence of transcriptomic dispersion or group-specific biological reprogramming.

### 2.3. Transcriptomic Divergence Identified via PCA

**Figure 3:**
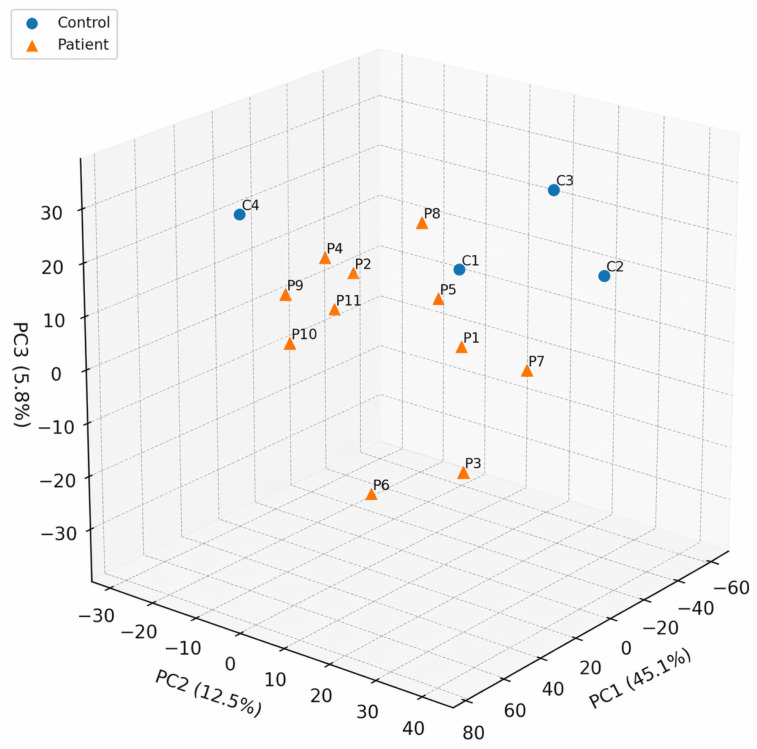
Principal component analysis (PCA) of peripheral blood RNA-seq samples based on log2(TPM + 1) values. Control samples are shown as blue circles and NIHL patients as orange triangles. Sample IDs are given. PCA is presented as an exploratory visualization of the overall sample structure, and the axes indicate the proportion of variance explained by each component. Color denotes group only and does not encode an additional quantitative variable.

Principal component analysis (PCA) was performed on log2(TPM + 1) values as an exploratory unsupervised approach to visualizing overall sample structure in the dataset. The first three principal components explained a substantial proportion of the total variance (PC1 = 45.1%, PC2 = 12.5%, and PC3 = 5.8%), enabling a dimensional reduction in the global expression matrix for descriptive assessment (Figure 3).

Visual inspection of the PCA plot suggested a partial shared structure between NIHL and control samples; however, complete separation between groups was not observed. One control sample appeared displaced relative to the remaining control samples, indicating within-group heterogeneity and limiting the strength of group-level interpretation based on PCA alone. Variability was also evident within the NIHL group, consistent with inter-individual heterogeneity in peripheral blood expression profiles. Accordingly, the PCA results should be interpreted as a descriptive visualization of global sample relationships rather than independent evidence of biological separation between groups. Sample IDs are given in Figure 3 for clarity.

### 2.4. Hierarchical Clustering of Selected High-Variance Genes

A heatmap was generated to visualize the expression profiles of the top 50 most variable genes across 15 peripheral blood RNA-seq samples, including 11 individuals with occupational noise-induced sensorineural hearing loss (SNHL; P1–P11) and four healthy controls (C1–C4), using log2-transformed TPM values (Supplementary Figure S1). To facilitate the comparison of relative expression levels across samples, expression values were standardized on a per-gene basis using z-score transformation. Hierarchical clustering was applied to both genes and samples using Euclidean distance and average linkage, allowing genes with similar expression profiles and samples with similar overall expression patterns to be grouped together.

For visualization purposes, samples were displayed according to phenotype, with SNHL cases arranged on the left and controls on the right. This presentation was intended to improve the interpretability of the heatmap and to facilitate the visual comparison of expression patterns between study groups. Across the selected high-variance genes, the heatmap demonstrated heterogeneity in relative expression levels among samples, with localized clusters of increased and decreased expression apparent across both the patient and control groups. These patterns suggest that a subset of highly variable transcripts contributes to inter-sample heterogeneity within the dataset.

Because the heatmap was constructed from genes selected on the basis of variance rather than statistical differential expression testing, the resulting clustering patterns should be interpreted as descriptive rather than inferential. In this context, the observed expression structure reflects relative variability among the selected transcripts and may be influenced by multiple factors, including biological heterogeneity, differences in blood cell composition, and normalization-related effects. Accordingly, the heatmap should be used to provide an overview of expression variability across samples rather than to establish definitive group-level transcriptomic separation.

### 2.5. Differential Gene Expression and Volcano Plot Analysis

Differential gene expression analysis was conducted using gene-level count data obtained from Salmon quantification outputs and analyzed with a DESeq2-based workflow suitable for bulk RNA-seq data with biological replicates. This approach models the mean–variance structure characteristic of count data and provides gene-wise estimates of expression change between the NIHL and control groups. Statistical significance was evaluated using Benjamini–Hochberg-adjusted *p*-values.

**Figure 4:**
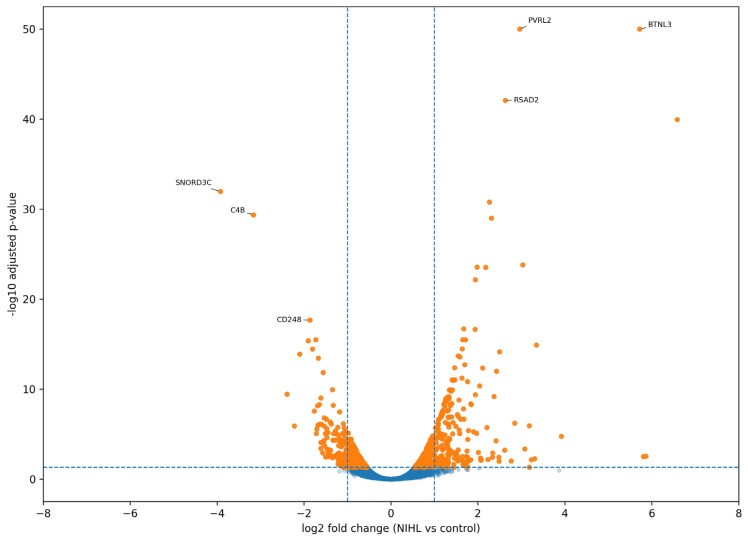
Volcano plot of differential gene expression based on DESeq2 analysis of count-derived RNA-seq data. The *x*-axis indicates log2 fold change (NIHL vs. control), and the *y*-axis indicates statistical significance as the −log10-adjusted *p*-value. Differential expression was assessed using DESeq2 with Benjamini–Hochberg multiple-testing correction. Selected representative genes are annotated for visualization. Points above the horizontal threshold line and beyond the vertical reference lines correspond to genes with larger effect sizes and stronger statistical support. Color coding indicates the differential expression significance categories of the plotted genes. Blue dotted points represent genes that did not meet the predefined differential expression significance threshold.

The overall distribution of differential expression results is shown in the volcano plot in Figure 4. In this representation, the *x*-axis denotes log2 fold change (NIHL vs. control), whereas the *y*-axis denotes −log10-adjusted *p*-value. Vertical dashed lines indicate reference fold change thresholds, and the horizontal dashed line marks the adjusted significance threshold corresponding to false discovery rate (FDR)= 0.05. Most transcripts were concentrated near the center of the plot, indicating limited expression differences between groups for the majority of genes. In contrast, a smaller subset of genes exhibited both larger effect sizes and stronger statistical support. Figure 4 therefore summarizes the complete DESeq2-based gene-level comparison between all NIHL cases and all controls rather than a restricted subset of genes.

Genes satisfying the predefined significance threshold are summarized in Table 2. This table presents the principal count-based differential expression results retained after multiple-testing correction, including gene identity, log2 fold change, mean normalized expression, test statistic, raw *p*-value, and adjusted *p*-value. The complete set of count-based differential expression results, including all tested genes and associated statistics, is provided in Supplementary Table S1. Additional supplementary analyses, including subsampling-based differentially expressed gene (DEG) stability assessment and exploratory classification performance evaluation, are provided in Supplementary Tables S2 and S3 and Supplementary Figure S2.

### 2.6. Functional Annotation of Differentially Expressed Genes

To explore the biological context of the differentially expressed genes identified in the count-based analysis, functional annotation was performed using curated databases including NCBI Gene, Reactome, and Gene Ontology (GO). The analysis focused on genes meeting the predefined significance threshold (FDR < 0.05) as reported in Table 2. Functional categorization of these genes indicated predominant representation of processes related primarily to immune response and host defense mechanisms. Several upregulated genes, including *RSAD2*, *IFIT1*, *IFI44L*, and *CMPK2*, are well-characterized interferon-stimulated genes (ISGs) associated with antiviral defense and innate immune activation. In addition, members of the defensin family (*DEFA1*, *DEFA3*, and *DEFA4*) showed increased expression, consistent with the activation of antimicrobial and neutrophil-associated pathways.

Genes related to antigen presentation and immune regulation were also observed, including *HLA-DRB1*, suggesting the involvement of adaptive immune processes. The upregulation of *GPR15*, a G protein-coupled receptor implicated in lymphocyte trafficking, further supports the presence of immune-cell redistribution or activation signatures in peripheral blood. In contrast, a smaller subset of downregulated genes was identified, including *SNORD3A* and other non-coding RNAs associated with ribosomal RNA processing. These observations may indicate alterations in RNA processing or nucleolar function; however, given the limited number of significantly downregulated transcripts, these findings should be interpreted cautiously.

Additional genes, such as *AREG* and *MDGA1*, are involved in cellular signaling and tissue-related processes, suggesting that transcriptional changes may extend beyond immune pathways. However, the overall functional profile of the significant DEGs was dominated by immune-related and interferon-associated signatures. Collectively, the functional annotation of count-based DEGs indicates that systemic immune activation and host defense pathways represent the most prominent transcriptomic features associated with occupational NIHL in peripheral blood.

## 3. Discussion

### 3.1. Overview of Differentially Expressed Genes

This study represents an exploratory assessment of peripheral blood transcriptomic alterations associated with occupational NIHL using a count-based RNA-seq analytical framework. After multiple-testing correction, a limited but statistically supported set of differentially expressed genes was identified, reflecting both the modest cohort size and the biological variability inherent in whole-blood transcriptomic data. Importantly, these findings are based on a DESeq2 reanalysis of gene-level count data with multiple-testing correction rather than on TPM-derived exploratory statistics.

The significant genes retained after count-based analysis were predominantly associated with immune activation and host defense. Among the most prominent findings in this dataset was the increased expression of interferon-stimulated genes, including *RSAD2*, *IFIT1*, *IFI44L*, and *CMPK2*. These genes are established components of type I interferon-responsive transcriptional programs and are closely associated with innate immune activation [17,18]. In parallel, members of the defensin family, including *DEFA1*, *DEFA3*, and *DEFA4*, were also upregulated in our analysis, demonstrating that antimicrobial and neutrophil-associated pathways are among the major features of the peripheral blood DEG profile [19,20,21].

Genes involved in antigen presentation and immune regulation were also represented among the significant transcripts. In particular, increased expression of *HLA-DRB1* suggests that major histocompatibility complex (MHC) class II-associated immune processes belong within the present DEG set; these pathways are central in antigen presentation and adaptive immune activation [22,23,24]. The significant gene set also included *GPR15*, a G protein-coupled receptor implicated in lymphocyte trafficking and immune-cell homing [25,26].

In contrast, a smaller subset of downregulated transcripts included non-coding RNAs such as *SNORD3A* and *SNORD3C*, which are associated with ribosomal RNA processing and nucleolar function. Small nucleolar RNAs are important regulators of rRNA maturation and ribosome biogenesis, and altered expression of these transcripts is consistent with the perturbation of RNA-processing-associated pathways [27,28,29,30].

In our study, RNA-processing-related transcripts represented a smaller component of the significant DEG set than the immune-associated genes. Overall, the differential expression landscape was dominated by interferon-associated, antimicrobial, and immune-regulatory transcripts, with more limited representation of RNA-processing-related genes.

### 3.2. Functional Interpretation of Significant Genes

Functional annotation of the genes retained after count-based differential expression analysis indicated that the principal biological signal in the dataset was centered on immune activation and host defense mechanisms. In particular, the concurrent differential expression of interferon-responsive genes (*RSAD2*, *IFIT1*, *IFI44L*, and *CMPK2*) and host-defense-related genes (*DEFA1*, *DEFA3*, and *DEFA4*) identified immune-associated pathways as the dominant component of the peripheral blood DEG profile [17,18,19,20,21]. Genes related to antigen presentation and immune regulation, including *HLA-DRB1* and *GPR15*, were also represented within the significant gene set [22,23,24,25,26].

Noise-induced cochlear injury has previously been linked to inflammatory and immune-related processes in experimental studies [8,13,31]. Within this broader context, our findings indicate that peripheral blood RNA-seq can detect a systemic transcriptomic pattern enriched in immune-associated genes in individuals with occupational NIHL. A smaller subset of downregulated transcripts, including *SNORD3A* and *SNORD3C*, was associated with RNA-processing-related functions [27,28,32], but this component was less prominent than the immune-related signal.

### 3.3. Comparison with Previous Transcriptomic Studies

Most previous transcriptomic investigations on NIHL have been conducted in cochlear tissues from animal models, with acute and subacute responses to acoustic trauma consistently implicating inflammatory, immune-related, and stress response pathways [10,33,34]. These studies have shown that noise exposure can induce complement activation, innate immune signaling, and broader transcriptional stress responses within the cochlea, thereby establishing inflammation as a central component of NIHL pathophysiology [8,10,33].

Against this background, in this study, we identified a peripheral blood DEG profile in which immune-associated transcripts represented the predominant differentially expressed component. In particular, the increased expression of interferon-responsive genes such as *RSAD2*, *IFIT1*, and *IFI44L* was compatible with the inflammatory and innate immune signatures previously described in cochlear transcriptomic studies after noise exposure [10,17,18]. The current dataset also included a defensin-associated component, indicating that host-defense-related pathways were represented alongside interferon-responsive transcripts [19,20,21].

Peripheral blood transcriptomes differ fundamentally from cochlear tissue transcriptomes in that they reflect circulating immune-cell populations and systemic physiological responses rather than local tissue-specific gene regulation. Blood-based profiles may therefore capture transcriptional consequences of chronic exposure, immune redistribution, or inflammatory adaptation that are not directly observable in cochlear samples. This distinction is particularly important in whole-blood RNA-seq, where the measured signal may be influenced by immune-cell composition as well as by cell-intrinsic transcriptional changes [35,36,37,38]. These results should therefore be interpreted as a peripheral blood signature shaped by both transcriptional regulation and the underlying blood cell composition.

In this regard, our findings complement rather than replicate prior tissue-based NIHL studies. Whereas animal cochlear transcriptomics have been instrumental in identifying local pathways of injury and repair, peripheral blood transcriptomics provide access to systemic responses associated with occupational noise exposure in human populations. A similar rationale has been applied in other exposure-related transcriptomic studies using whole blood, where biologically meaningful systemic signals have been detected even when the primary target tissue is not accessible [12,39].

Our findings extend previous NIHL transcriptomic research by identifying a peripheral immune-related transcriptional signature in whole blood. Although these systemic signals do not directly reflect cochlear tissue-specific transcriptional events, they provide a practical and biologically informative complement to tissue-based studies in human exposure settings. In this context, the current dataset indicates that peripheral blood RNA-seq can detect a coherent immune-related signal in occupational NIHL despite the absence of direct cochlear tissue access. The biological origin and temporal persistence of peripheral immune-related transcriptomic signals after established NIHL remain incompletely characterized in humans; as a result, these findings should be considered exploratory.

### 3.4. Implications for Biomarker Discovery

The identification of statistically supported differentially expressed genes in peripheral blood supports the use of transcriptomic profiling as a framework for investigating candidate biomarkers of occupational NIHL. In this study, the candidate gene set was dominated by interferon-associated, host-defense-related, and immune-regulatory transcripts, indicating that the most robust peripheral blood signals were centered on immune-associated processes [17,18,19,20,21,22,23,24,25,26].

Importantly, these genes were retained after DESeq2-based analysis with multiple-testing correction and therefore define a focused candidate set based on statistically supported differential expression rather than exploratory TPM-based comparisons alone. At the same time, the translational implications of these findings should be interpreted within the constraints of our study design. The cohort size was limited, and although the use of a count-based framework with multiple-testing correction increases the statistical rigor of the analysis, the resulting DEG set remains exploratory in nature. This constraint is especially relevant for the control group, which was small and may not have fully captured the range of peripheral blood transcriptomic variation in non-exposed individuals. A supplementary subsampling-based stability assessment using gene-level Salmon-derived count estimates further indicated that several leading candidate genes, including *PVRL2*, *BTNL3*, *RSAD2*, *MDGA1*, *IFIT1*, *GPR15*, *SNORD3A*, and *CD248*, retained stable effect-direction consistency across repeated reduced-sample iterations, whereas candidates such as *HLA-DRB1*, *DEFA1*, and *DEFA3* were more sensitive to sample perturbation. These findings reinforce the exploratory interpretation of the present DEG set and indicate that larger independent cohorts will be necessary to determine which candidates remain robust under broader sampling conditions.

In addition, whole-blood transcriptomic data are influenced by variations in leukocyte and erythroid cell composition, which may affect differential expression results if not explicitly modeled [35,36,37,38]. This consideration is particularly relevant in our cohort, because clinical hematologic data, such as complete blood counts with differentials, were not available for integration into the transcriptomic analysis. In addition, one participant in the NIHL group had asthma, a chronic inflammatory condition that may have contributed to individual-level variation in peripheral blood gene expression. Because the cohort included middle-aged adults with heterogeneous occupational exposure histories, age-related contributions to the observed hearing profiles cannot be fully excluded at the individual level. However, the dominant group-level audiometric pattern remained consistent with high-frequency NIHL.

For these reasons, the genes identified here should not be considered validated biomarkers at this stage. Rather, they define a biologically plausible candidate set for future validation studies. Biomarker development in transcriptomics generally requires staged evaluation, including analytical validity, reproducibility across independent cohorts, assessment of biological and clinical relevance, and, ultimately, demonstrations of clinical utility [40,41,42,43,44].

Future investigations should therefore incorporate larger and independently recruited populations, more balanced control sampling, and replication across different occupational settings. The integration of cell-composition-aware analytical strategies, together with clinical hematologic data such as complete blood counts with differentials, would improve the interpretation of peripheral blood transcriptomic signals in relation to underlying immune-cell distributions. Exposure-stratified analyses and more detailed characterization of occupational noise profiles would also help determine whether distinct acoustic environments are associated with different peripheral transcriptomic patterns. In addition, longitudinal study designs will be key in determining the temporal stability and biomarker relevance of these signals after established NIHL.

Multi-gene signatures may ultimately be more informative than single transcripts alone. Integrated host-response panels have shown improved robustness and portability in other transcriptomic diagnostic settings, suggesting that composite signatures may offer greater potential than any individual DEG in isolation [45]. Our study therefore constitutes an initial step toward defining peripheral blood transcriptomic candidates associated with occupational NIHL. The significant genes prioritized in this analysis provide a concrete starting point for targeted validation in independent cohorts and with orthogonal molecular assays. An additional exploratory machine-learning-based classification analysis using filtered log2(TPM + 1) expression profiles showed preliminary discriminatory capacity between NIHL and control samples under leave-one-out cross-validation, with an accuracy of 0.867, balanced accuracy of 0.830, and ROC-AUC of 0.773 in the evaluated model configuration (Supplementary Figure S2, Supplementary Table S3). Given the limited cohort size and lack of external validation, these results should be interpreted as proof-of-concept findings rather than as definitive evidence of diagnostic performance. The diagnostic relevance of these exploratory classification findings will require evaluation in independent external cohorts, and future studies should extend this framework by incorporating supervised classification models and formal feature selection strategies. From a computational perspective, future transcriptomic biomarker studies in occupational NIHL may also benefit from recent advances in graph-based biomedical modeling for complex biological prediction tasks [46]. Recent reviews further indicate that Transformer–graph neural network integration and multimodal pre-training frameworks are becoming increasingly important for biological representation learning and predictive modeling in data-rich biomedical settings [47]. In addition, current methodological surveys in computational genomics are highlighting the growing importance of graph-based mapping and representation strategies for complex biological data structures, which may also inform future transcriptomic analysis pipelines as datasets become larger and more heterogeneous [48]. Related work also supports the use of integrated Transformer and graph-attention-based predictive frameworks as emerging computational strategies for biomolecular interaction modeling [49].

## 4. Materials and Methods

### 4.1. Study Participants

The study cohort consisted of 15 male participants, including 11 patients diagnosed with occupational noise-induced bilateral SNHL and 4 healthy controls without clinical signs of hearing loss. Patients were recruited from the outpatient clinic of Istanbul Yedikule Chest Diseases and Chest Surgery Education and Research Hospital, and the study procedures were conducted in coordination with the Department of Otolaryngology (Ear, Nose and Throat), Istanbul Faculty of Medicine, where audiometric testing was performed. All SNHL patients had a history of long-term occupational noise exposure and were employed in high-risk fields such as the automotive industry and textile manufacturing. Audiometric evaluation confirmed bilateral SNHL in all patients.

The control group consisted of four healthy male volunteers without documented hearing impairment or chronic illness. Control participants had no history of occupational noise exposure and showed normal audiometric findings. The age range of the SNHL group was 39–54 years, whereas that of the control group was 35–52 years. Ethical approval for this study was obtained from the Clinical Research Ethics Committee of Istanbul Medical Faculty on 13 August 2025 (Approval No: E-29624016-050.99-3521284). All procedures were conducted in accordance with the Declaration of Helsinki. Written informed consent was obtained from all participants prior to enrollment. Complete blood counts with differentials and additional systemic inflammatory laboratory parameters were not incorporated into our study design. As a result, potential between-subject variation in circulating immune-cell composition or background inflammatory status could not be directly controlled at the clinical sampling stage.

### 4.2. Total RNA Isolation

Total RNA was extracted from whole-blood samples using the QIAamp RNA Blood Mini Kit (Cat. No. 52304; Qiagen, Hilden, Germany), according to the manufacturer’s protocol optimized for human blood. The protocol involved selective lysis, protein digestion, and spin-column purification. RNA concentration and purity were assessed spectrophotometrically using a Nanodrop™ ND-1000 (Thermo Fisher Scientific, Wilmington, DE, USA), measuring A260/A280 and A260/A230 ratios. RNA integrity was evaluated using the Agilent 2100 Bioanalyzer (Agilent Technologies, Santa Clara, CA, USA) and reported as the RNA Integrity Number (RIN). Only samples with RIN ≥ 7.0 and total RNA yield ≥ 100 ng were advanced to sequencing.

### 4.3. Library Preparation and RNA Sequencing

RNA-seq libraries were constructed using Illumina-compatible RNA library kits (Illumina Inc., San Diego, CA, USA) following standard mRNA-seq workflows, including messenger RNA (mRNA) enrichment, fragmentation, complementary DNA (cDNA) synthesis, end repair, adapter ligation, and polymerase chain reaction (PCR) amplification. Sequencing was performed using the Illumina DRAGEN RNA Pipeline (Illumina Inc., San Diego, CA, USA), which incorporates read trimming, alignment (via STAR), and quantification. Paired-end sequencing (2 × 100 bp) was conducted on an Illumina platform (Illumina Inc., San Diego, CA, USA), ensuring a minimum of 25 million reads per sample. The DRAGEN platform enabled automated and reproducible preprocessing of raw data into high-quality FASTQ and quantification outputs.

### 4.4. Transcript Quantification and TPM Calculation

Transcript quantification was performed using Salmon v1.9.0 (Patro et al., Center for Computational Biology, Johns Hopkins University, Baltimore, MD, USA) in quasi-mapping mode against the human reference transcriptome (GENCODE release 42, GRCh38 assembly) [17]. This lightweight approach provided fast and accurate abundance estimation while accounting for sequence-specific bias. Transcript-level estimates were collapsed to the gene level using tximport in R [18]. TPM values were retained for exploratory visualization only, whereas gene-level count estimates were imported into DESeq2 for formal differential expression analysis.

### 4.5. Data Normalization and Filtering

TPM values were transformed using log2(TPM + 1) to reduce skewness and improve visualization of expression distributions. Genes with zero expression in more than 50% of samples were excluded from downstream exploratory analyses to minimize background noise and reduce the influence of sparsely expressed features. The log2(TPM + 1)-transformed dataset was used for descriptive visualizations, including boxplots, PCA, and heatmap generation, whereas formal differential expression analysis was performed separately on gene-level count data using DESeq2. No TPM-derived values were used for formal statistical testing of differential expression.

### 4.6. Statistical and Bioinformatic Analysis Workflow

After log2 transformation and filtering of TPM values, downstream analyses were conducted using a structured statistical and bioinformatics pipeline. For exploratory analysis, unsupervised clustering methods were applied to visualize sample-to-sample variation. These included boxplot generation to examine overall expression distribution and principal component analysis (PCA) using the scikit-learn package, version 1.8.0, in Python, version 3.14.4, with samples color-coded by experimental group. PCA was conducted on z-score-standardized expression values and visualized as an exploratory approach to assessing overall sample structure, within-group heterogeneity, and potential outliers. PCA and visualization were implemented in Python using scikit-learn, matplotlib, and related plotting utilities. Hierarchical clustering and heatmap generation were performed in Python, version 3.14.4, using Seaborn, version 0.13.2, and SciPy, version 1.17.1.

Differential expression analysis was performed using gene-level count data derived from Salmon quantification outputs. Transcript-level abundance estimates were imported with tximport and analyzed in R using DESeq2, which models count data according to a negative-binomial distribution and is appropriate for RNA-seq experiments with biological replicates. Differential expression analysis was performed using the study group (NIHL vs. control) as the design factor. Unless otherwise stated, DESeq2 default procedures for dispersion estimation, model fitting, and independent filtering were applied. No machine-learning-based feature selection or classifier training was incorporated into the primary differential expression workflow. Exploratory classification analyses were conducted separately as supplementary computational assessments. Size-factor normalization was applied within the DESeq2 framework to account for sequencing depth and compositional differences between samples. Differential expression between the SNHL (P1–P11) and control (C1–C4) groups was assessed using the DESeq2 Wald test, and *p*-values were adjusted for multiple testing using the Benjamini–Hochberg false discovery rate (FDR) procedure. Adjusted *p*-values were used to define statistical significance. A volcano plot was generated from the full gene-level differential expression output obtained from DESeq2 by comparing the NIHL group (P1–P11) against the control group (C1–C4). Each point represents one tested gene, positioned according to log2 fold change and adjusted *p*-value. As an additional sensitivity analysis, a subsampling-based stability assessment was performed using gene-level Salmon-derived count estimates. Repeated reduced-sample iterations were generated through the random resampling of 9 out of 11 NIHL cases and 3 out of 4 controls, and effect-direction consistency was evaluated across iterations for the principal candidate genes. As an exploratory computational assessment, supervised classification analyses were also performed on filtered log2(TPM + 1) gene expression profiles using leave-one-out cross-validation. Candidate features were evaluated within the training folds, and classification performance was assessed using accuracy, balanced accuracy, and receiver operating characteristic area under the curve (ROC-AUC). These analyses were intended as proof-of-concept evaluations and not as formal diagnostic model validation. The resulting performance metrics and ROC analysis are provided in Supplementary Figure S2 and Supplementary Table S3. The ROC analysis shown in Supplementary Figure S2 was derived from the leave-one-out cross-validated logistic regression configuration.

Hierarchical clustering and heatmap generation were performed using the Seaborn package, version 0.13.2, in Python, version 3.14.4. The top 50 most variable genes were visualized as a descriptive heatmap of relative expression patterns across samples. Gene-level interpretations were informed by functional annotations from public databases including GeneCards [(https://www.genecards.org, accessed on 14 December 2025)] [50], KEGG [(https://www.genome.jp/kegg, accessed on 14 December 2025)] [51], Reactome [(https://reactome.org, accessed on 14 December 2025)] [52], and NCBI Gene [(https://www.ncbi.nlm.nih.gov/gene, accessed on 14 December 2025)] [53], providing insight into molecular pathways such as ribosomal RNA processing, immune activation, and stress response mechanisms.

### 4.7. Quality Control Assessment

Sequencing quality metrics were assessed using MultiQC. Sample-level indicators including total input reads, mapping performance, duplication rate, properly paired reads, and GC content were reviewed across all samples. Overall, the quality control (QC) metrics were broadly comparable across the dataset and did not indicate major technical failures that would preclude downstream analysis. These QC assessments were used to support downstream interpretation but were not considered to be sufficient for distinguishing biological from technical sources of variation in unsupervised analyses.

## 5. Conclusions

This study represents an exploratory analysis of peripheral blood transcriptomic alterations associated with chronic occupational noise exposure and sensorineural hearing loss. The signals observed were dominated by immune-related transcripts, including interferon-associated and host-defense-related genes, with more limited evidence of alterations in RNA-processing-related pathways. These findings suggest that occupational NIHL may be associated with detectable peripheral blood transcriptional changes; however, the results require validation in larger independent cohorts before biological or clinical utility can be established.

## Figures and Tables

**Table 1:** Hearing thresholds at different frequencies in noise-induced hearing loss (NIHL) and control groups.

**Hearing Loss Group**	0.5 kHz	0.75 kHz	1 kHz	2 kHz	3 kHz	4 kHz	6 kHz	8 kHz
Ear	M (SD)	M (SD)	M (SD)	M (SD)	M (SD)	M (SD)	M (SD)	M (SD)
Right Ear	29.5 (17)	30 (18)	30.5 (18)	38.2 (17.5) *	40.5 (18.5) *	58.2 (19.5) *	55.9 (21.5) *	61.8 (21) *
Left Ear	35 (25.5)	35 (23)	35 (22)	46.5 (20.5) *	48.6 (19.5) *	52.9 (18.5) *	56.8 (17.5) *	60.5 (20.5) *
Control Group								
Right Ear	16.5 (2.5)	17 (3)	15.5 (2.5)	16 (2.5)	18.5 (2.5)	15.3 (3)	15.5 (2.5)	13.5 (3.5)
Left Ear	15 (3)	16.5 (2.5)	14.5 (3.5)	15.5 (2.5)	15.5 (3.5)	16 (2.5)	14.5 (3.5)	14.5 (2.5)

M, mean; SD, standard deviation; * *p* < 0.05.

**Table 2:** Differentially expressed genes (FDR < 0.05) identified via DESeq2 analysis of gene-level RNA-seq counts.

Gene	log2 FC	baseMean	Stat	*p*-Value	Padj	Direction
*PVRL2*	2.959226349	921.0828637	15.57127	1.14121 × 10^−54^	9.96718 × 10^−51^	Up in NIHL
*BTNL3*	5.719300414	208.5150034	15.56437	1.27108 × 10^−54^	9.96718 × 10^−51^	Up in NIHL
*RSAD2*	2.62835875	2160.087404	14.32374	1.55525 × 10^−46^	8.13035 × 10^−43^	Up in NIHL
*MDGA1*	6.584065035	202.1590085	13.95599	2.89265 × 10^−44^	1.13414 × 10^−40^	Up in NIHL
*SNORD3C*	−3.926774262	16.05996311	−12.5588	3.55865 × 10^−36^	1.11621 × 10^−32^	Down in NIHL
*DEFA3*	2.267184927	1565.960289	12.32998	6.24654 × 10^−35^	1.63274 × 10^−31^	Up in NIHL
*C4B*	−3.161070578	21.05635436	−12.0515	1.90449 × 10^−33^	4.26687 × 10^−30^	Down in NIHL
*HLA-DRB1*	2.308357584	477.2244397	11.96762	5.25146 × 10^−33^	1.02948 × 10^−29^	Up in NIHL
*NEBL*	3.029701947	79.88661117	10.92276	8.97287 × 10^−28^	1.56357 × 10^−24^	Up in NIHL
*IFIT1*	1.977990503	2403.58395	10.86185	1.75155 × 10^−27^	2.74696 × 10^−24^	Up in NIHL
*COMMD3-BMI1*	2.178359375	267.688771	10.84761	2.04719 × 10^−27^	2.91873 × 10^−24^	Up in NIHL
*IFI44L*	1.938160008	1321.650366	10.54605	5.29766 × 10^−26^	6.92360 × 10^−23^	Up in NIHL
*CD248*	−1.863517207	96.3566028	−9.52087	1.71736 × 10^−21^	2.07180 × 10^−18^	Down in NIHL
*DEFA1*	1.674775909	6704.381704	9.274296	1.78796 × 10^−20^	2.00290 × 10^−17^	Up in NIHL
*DEFA4*	1.93080361	164.893605	9.254853	2.14526 × 10^−20^	2.24294 × 10^−17^	Up in NIHL
*GPR15*	1.710250532	392.0734941	8.9493	3.57744 × 10^−19^	3.21260 × 10^−16^	Up in NIHL
*CMPK2*	1.649691478	966.5465735	8.946579	3.66669 × 10^−19^	3.21260 × 10^−16^	Up in NIHL
*SNORD3A*	−1.729547106	113.7252421	−8.94596	3.68722 × 10^−19^	3.21260 × 10^−16^	Down in NIHL
*NRCAM*	−1.903394528	43.13954713	−8.9084	5.17766 × 10^−19^	4.27375 × 10^−16^	Down in NIHL
*AREG*	3.343834757	38.68440783	8.782264	1.60217 × 10^−18^	1.25634 × 10^−15^	Up in NIHL

## Data Availability

The original contributions presented in this study are included in the article/Supplementary Material. Further inquiries can be directed to the corresponding author.

## Supplementary Materials

The following supporting information can be downloaded at: https://www.mdpi.com/article/10.3390/ijms27104182/s1.
