# Supplementary figures and images for: Transcriptomic Traces of Noise Exposure in Hearing Loss and Systematic Identification of Biomarker Candidates at the Molecular Scale
Source: Int J Mol Sci. 2026 May 8;27(10):4182. doi: 10.3390/ijms27104182 (PMC13206948; doi:10.3390/ijms27104182)

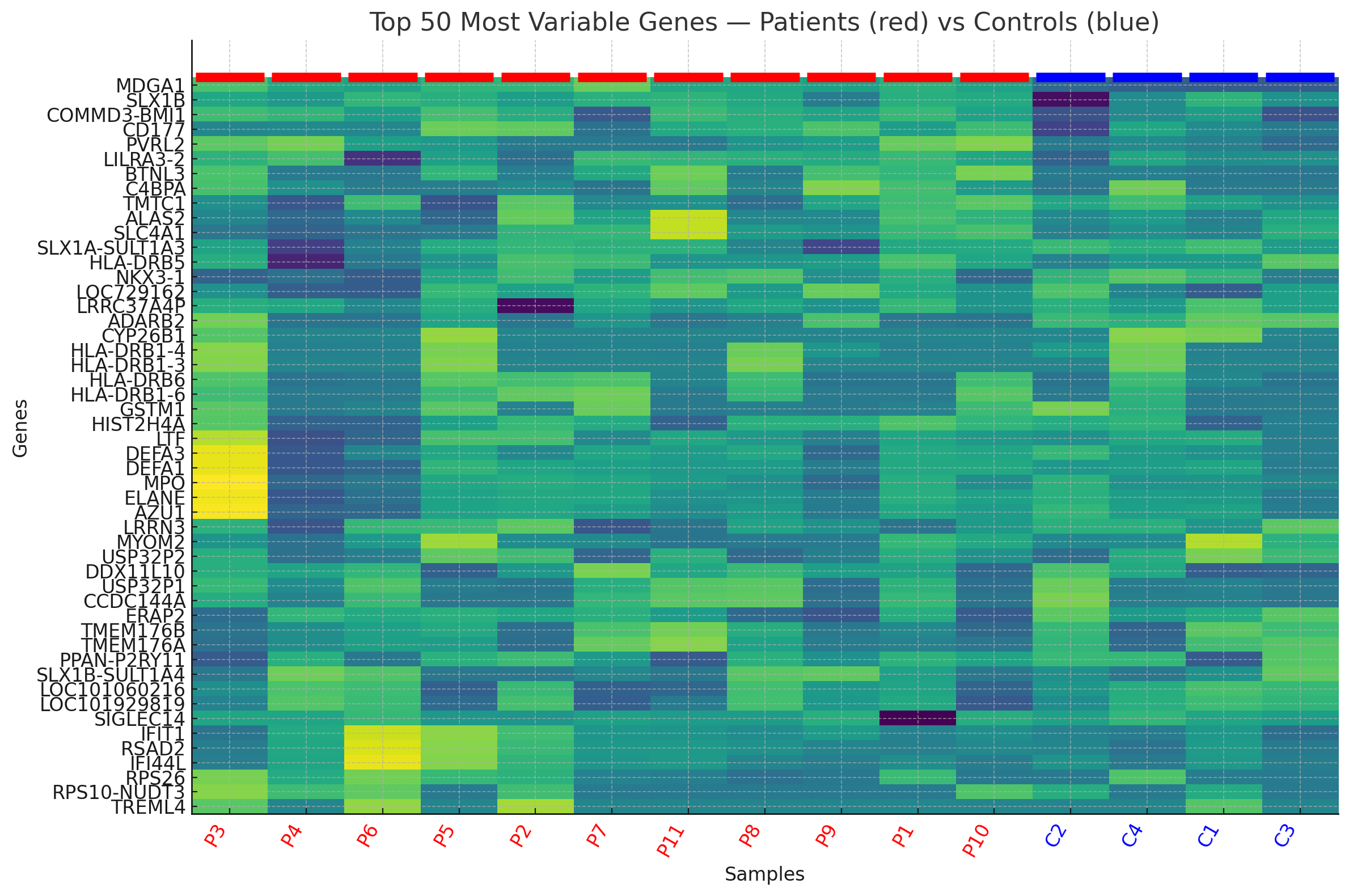

Supplement: Supplementary file 1 [file ijms-27-04182-s001.zip › Supplementary Figure S1.png]

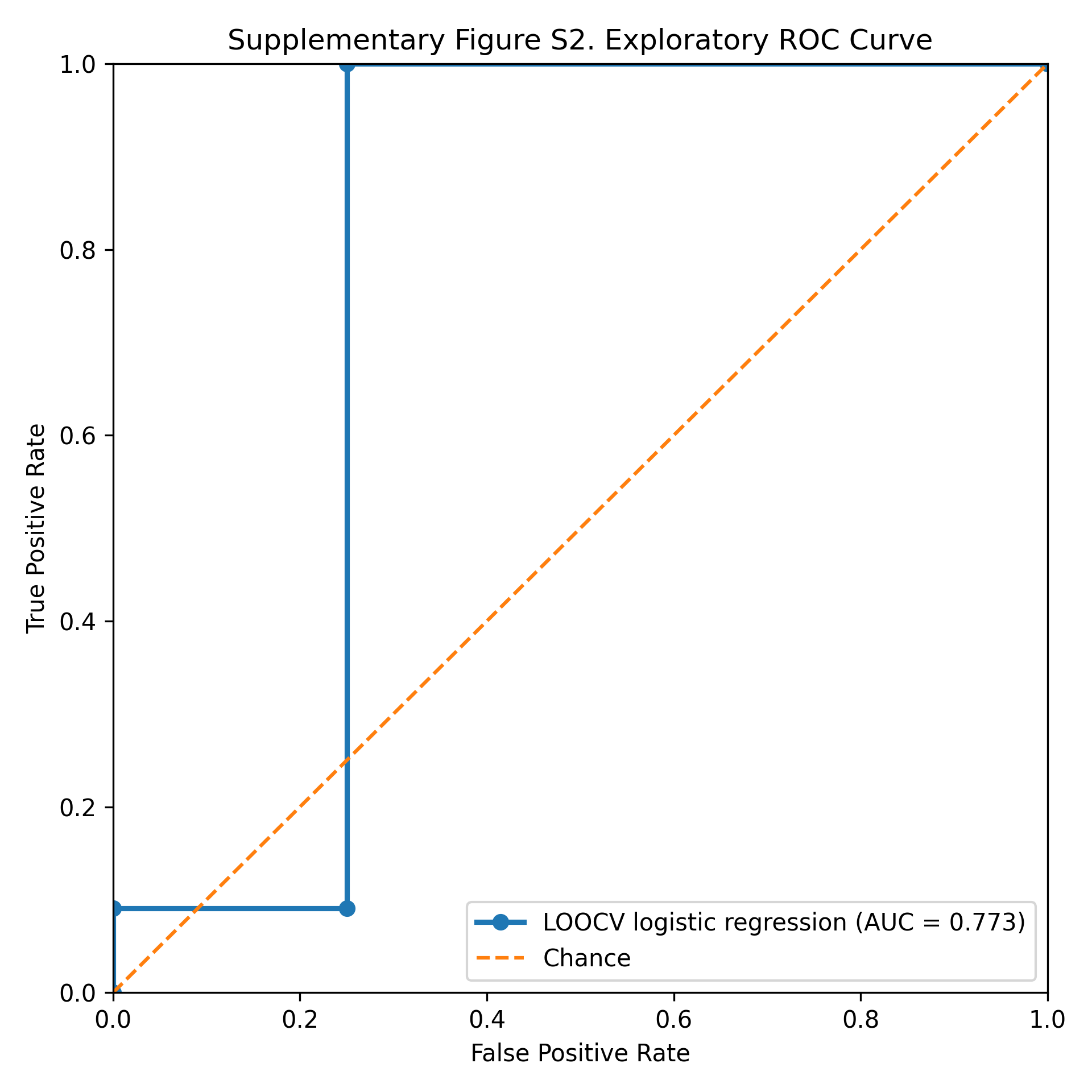

Supplement: Supplementary file 1 [file ijms-27-04182-s001.zip › Supplementary Figure S2.png]
